# Expertise about herbs and dietary supplements among diverse health professionals

**DOI:** 10.1186/1472-6882-6-15

**Published:** 2006-04-28

**Authors:** Kathi J Kemper, Paula Gardiner, Jessica Gobble, Charles Woods

**Affiliations:** 1 Departments of Pediatrics and Public Health Sciences, Wake Forest University School of Medicine, Medical Center Blvd., Winston-Salem, NC, USA; 2 Division for Research and Education in Complementary and Integrative Medical Therapies, Harvard Medical School, Boston, MA, USA; 3 Northwest Area Health Education Center, Wake Forest University School of Medicine, Winston-Salem, NC, USA

## Abstract

**Background:**

Herbs and other dietary supplements are among the most commonly used complementary medical therapies. However, clinicians generally have limited knowledge, confidence and communication about herbs and dietary supplements (HDS). We compared diverse clinicians' expertise about HDS to better target future curricula.

**Methods:**

We conducted a cross-sectional survey of physicians, pharmacists, nurses, dietitians and trainees in these professions prior to e-curriculum about HDS in 2004–2005. The survey had 28 questions about knowledge, 19 questions about their confidence and 11 questions about their communication practices about HDS.

**Results:**

Of the 1,268 participants, 25% were male; the average age was 40 years. Mean scores were 66% correct for knowledge; 53/95 on the confidence scale and 2.2 out of possible 10 on the communication practices scale. On average, scores were lowest for those who used fewer HDS; and trainees and nurses compared with physicians, pharmacists and dietitians (P<0.01 for all comparisons).

**Conclusion:**

Clinicians have moderate levels of knowledge and confidence, but poor communication skills about HDS. Future curricula about HDS should target nurses, students, practitioners and those not currently using HDS. Research is needed to determine the most cost-effective educational strategies for diverse health professionals.

## Background

Herbs and dietary supplements (HDS) are the most commonly used complementary or alternative medical (CAM) therapies in the US besides prayer [1]. In national telephone surveys of US adults, the prevalence of using HDS increased from 14% in 1998–1999 to 19% in 2002; it doubled in those 65 years and older [2]. Annual expenditures for HDS exceed $4 billion annually [2-6]. Substantial numbers of all patient groups report using HDS, particularly women and those with chronic or recurrent illnesses who also receive care by conventional health care professionals [7-15]. Despite the high prevalence of patient use, fewer than half of patients who use CAM typically discuss it with their clinician [16]; in part, this is because health care professionals do not consistently inquire about or record patients' use of HDS, and in part because patients do not perceive health care professionals as particularly knowledgeable about HDS [17-19].

In a previous survey, we identified substantial room for improvement in clinician knowledge, confidence and communication practices about HDS [20]. These findings were confirmed in a study of resident trainees [21]. Objective review of medical records bears out the poor communication practices noted in self-report surveys; for example, in a chart review of 67 patients hospitalized for asthma treatment, no charts documented use of HDS, although interviews indicated that over 40% of such patients used HDS as an asthma treatment [18].

Given the great need for professional education on this topic, we wished to understand differences among health professionals to better target future educational programs. Based on previous research, we had three hypotheses: 1) there would still be substantial room for improvement in knowledge, confidence and communication practices in all groups; 2) practicing clinicians would have greater confidence than trainees; and 3) higher personal use of HDS would be associated with higher scores.

## Methods

We surveyed a cross sectional convenience sample of clinicians prior to their enrollment in an on-line course about HDS offered through the Northwest Area Health Education Center (NW AHEC) of Wake Forest University School of Medicine (WFUSM) and a part of the North Carolina AHEC system in Fall, 2004 and Spring, 2005. The surveys were completed prior to receiving curriculum.

Participants were eligible if they were in one of four professional groups: physicians (including physician assistants, PA), nurses (including advanced practice nurses), pharmacists or dietitians or trainees in one of these four health professions. Trainees included students, interns, residents and post-doctoral fellows in any of the four professional groups. Subjects were excluded if they reported using the internet less than twice weekly. Enrollment occurred online.

### Recruitment

In the summer of 2004 the NW AHEC sent emails to approximately 27,000 individuals in its state-wide continuing education (CE) database informing them about the on-line curriculum. Emails were also sent to department chairs for medicine, nursing, pharmacy and nutrition at North Carolina (NC) health professions schools. Finally, invitations via email were sent to colleagues, personal contacts and professional list-serve groups by the Principal Investigator (PI). Overall, we estimate that a total of approximately 29,000 emails were sent directly by course faculty and staff advertising the curriculum. "Viral" marketing (i.e., forwarding of the original emails) also occurred but was not formally monitored. Approximately 500 flyers were also distributed at various continuing education activities.

Recruitment was similar for Spring, 2005. In addition, emails were sent to the director of South Carolina (SC) AHEC with requests to forward the emails to their distribution lists. We also sent emails to faculty at SC schools of nursing, pharmacy, nutrition, and medicine that were listed on the Internet, and asked them to forward information about the course to interested faculty and staff. Emails were also sent to the WFUSM Alumni association and listservs for the Ambulatory Pediatric Association and the Society of Teachers of Family Medicine. We also sent a notice to the listserv for the 27 members of the Consortium of Academic Health Centers for Integrative Medicine (CAHCIM); two of these CACHIM-affiliated medical schools promoted the program to their students by email. Brochures were mailed to 19,000 persons on the NC AHEC health professions database and the WFUSM Continuing Medical Education (CME) database who did not have email addresses listed.

Participants registered through the NW AHEC website; their registration data, including demographic information was imported into the AHEC database. Following registration, participants completed the baseline questionnaire on-line. For the fall, 2004 curriculum, 321 were eligible and completed the baseline; for spring, 2005, 947 completed the baseline and were eligible for analysis, resulting in a total sample of 1268 eligible participants.

### Survey instrument

The survey instrument was based on the tool used in our earlier pilot study [20]. In addition to demographic data, we asked about participants' profession, whether they were students or another type of trainee, whether they had seen any patients in the 30 days prior to the survey and about their own use of herbs and dietary supplements, providing a list of approximately 100 possible HDS. Based on the pilot study, we modified and increased the number of items for the knowledge questions, confidence and communication practice scales.

*Knowledge scores*were generated as percent of the knowledge questions answered correctly. The knowledge questions included items about the use and safety of commonly used herbs and dietary supplements such as green tea, St. Johns wort, ephedra, saw palmetto, ginkgo, black cohosh, folate, chromium, fish oil and glucosamine. There were 13 true-false (TF) questions and 15 MC questions. Scores could range from 0 to 100% correct.

A *confidence scale*score was derived from responses to 19 Likert-type questions (strongly disagree, disagree, neutral/not sure, agree, strongly agree) such as "I feel confident responding to patients' questions about HDS." [see Additional file 1] Each item was scored 1 (strongly disagree) to 5 (strongly agree), with a minimum score of 19 and maximum of 95. The Cronbach alpha reliability statistic was 0.96 for the confidence scale.

Eleven items were used to create a *communications practices scale*for those who had seen patients within the past 30 days. [See Additional file 1] Nine items asked for responses in terms of 10% increments from 0 to 100% (e.g., "In the past 30 days, in what percentage of your clinical encounters have you discussed with a patient or family the use of HDS?"). Two questions were in yes-no format regarding whether in the past 30 days, they had: 1) cautioned any patient about potential hazards of HDS; and 2) discussed a question about HDS with any colleagues. These items were combined into a *communications practices scale*, with the first nine items scored as a proportion corresponding to the percentage chosen (0.0 to 1.0) and the two yes-no items scored as 0.5 for yes and 0 for no. The possible range of scale scores was 0 to 10. The Cronbach alpha reliability statistic was 0.84 for communications practices scale.

### Analysis

Descriptive statistics were generated using means and standard deviations for normally distributed data and medians for non-normally distributed data. Two way comparisons were tested by Chi-square for nominal and categorical data, using t-tests for normally distributed data and non-parametric tests such as Mann-Whitney U tests and Kruskal Wallis tests for non-normally distributed variables. Backward conditional multiple regression analysis was performed to assess the relative importance of individual factors in terms of association with selected outcomes measures. Analyses were performed using SPSS 13.0 (SPSS Inc., Chicago, IL).

This study was approved as "exempt" as an educational research project by the Wake Forest University School of Medicine Institutional Review Board.

## Results

Of the 1268 eligible participants, the average age was 40 years and 25% were male (Table 1). Overall, 57% of participants described themselves as being in practice or on faculty; the rest were trainees, such as students, interns, residents or post-doctoral fellows. Most participants (N = 853) reported having seen a patient in the 30 days prior to participation. Most (85%) respondents used herbs and dietary supplements (HDS) themselves, reporting a median use of four HDS daily. The most frequently used HDS were: multivitamins (64%), calcium (39%), B vitamins (33%), C vitamins (33%), green tea (26%), fish oil (26%), vitamin E (23%), flax seed (18%) and vitamin D (16%).

**Table 1:** Demographic, knowledge and practice characteristics of subjects.

**Characteristic**	**Total**	**Fall, 2004**	**Spring, 2005**	**P value**
N	1268	321	947	
Age (mean)	40.3 ± 12.9	43.5 ± 11.4	39.3 ± 13.2	<.001*
Gender (% male)	321 (25%)	62 (19%)	259 (27%)	.005†
Race				.18†
African American	57 (4.5%)	14 (4.4%)	43 (4.5%)	
Asian/Pacific Islander	96 (7.6%)	15 (4.7%)	81 (8.6%)	
Caucasian	1052 (83%)	276 (86%)	776 (82%)	
Native American/Alaskan Native	3 (0.2%)	0	3 (0.3%)	
Declined	60 (4.7%)	16 (5.0%)	44 (4.6%)	
Ethnicity (% Latino)	41 (3.2%)	7 (2.2%)	34 (3.6%)	.37†
Professional Group				<.001†
Physician/PA	374 (29.5%)	107 (33.3%)	267 (28.2%)	
Nurse	296 (23.3%)	92 (28.7%)	204 (21.5%)	
Nutritionist	150 (11.8%)	62 (19.3%)	88 (9.3%)	
Pharmacist	58 (4.6%)	32 (10.0%)	26 (2.7%)	
Student	390 (30.8%)	28 (8.7%)	362 (38.2%)	
Used HDS in the past week	1079 (85.1%)	259 (80.7%)	820 (86.6%)	.013†
Number of HDS used in the past week	5.5 ± 6.3 median 4	4.4 ± 5.0 median 3	5.9 ± 6.7 median 4	<.001*
NC resident [yes]	599 (47.2%)	217 (67.6%)	382 (40.3%)	<.001†
Faculty/In Practice	727 (57.3%)	251 (78.2%)	476 (50.3%)	<.001†
Had seen patients in past 30 days? [yes]	853 (67.3%)	247 (76.9%)	606 (64.0%)	<.001†
Knowledge Scores (% of items answered correctly)	65.8 ± 10.7	68.8 ± 10.8	64.8 ± 10.4	<.001*
Confidence Scale (range 19–95)	52.5 ± 18.2 n = 1201	66.4 ± 15.8 n = 321	47.5 ± 16.3 n = 880	<.001*
Communication Practices Scale ‡ (range 0–10)	2.20 ± 1.92 n = 852	1.88 ± 1.72 n = 247	2.33 ± 1.99 n = 606	.002*

* Determined using Mann-Whitney U test

† Determined using Chi square method with continuity correction for 2 × 2 tables.

‡ Determined for those who had seen patients in the past 30 days.

The number of students (N = 390) is less than the number of trainees (non-faculty/practice clinicians, N = 540) because trainees include residents, fellows, and post-doctoral fellows in the four professions.

Participants in fall and spring semesters differed in several ways (Table 1). Spring participants were less likely to have seen a patient in the 30 days prior to enrollment (64% vs. 77%, P<0.001) and less likely to be on faculty or in practice (50% vs. 78%, P<.001) than were fall participants. Spring participants were significantly more likely to be male, to be students, and to be from outside North Carolina.

Overall, respondents answered an average of 65.8% of the knowledge test items correctly (Table 2). Scores were significantly lower for students than any professional group; nurses had lower scores than other professionals. Scores were significantly lower for those who had not seen patients in the past 30 days than for those who had; and highest for those using = 9 supplements daily. Because some of these variables were likely related, multiple regression analyses were performed (see below).

**Table 2:** Knowledge and Confidence scores by demographic and practice characteristics.

**Characteristic**	**% Knowledge Items Answered Correctly**	P value*	**Confidence Scale (Range 19,95)**	P value*
Overall mean	65.8 ± 10.7		**52.5**± 18.2	
Profession (note 1†)		<.001		<.001
Physician/PA	70.3 ± 9.8		55.5 ± 18.5	
Nurse	64.9 ± 9.3		54.2 ± 18.5	
Nutritionist	68.1 ± 9.4		59.2 ± 17.3	
Pharmacist	70.7 ± 10.2		59.8 ± 15.8	
Student	60.5 ± 10.5		44.6 ± 15.7	
Gender		.39		.30
Males	65.3 ± 11.3		51.5 ± 18.2	
Females	66.0 ± 10.4		52.9 ± 18.2	
Age (years) (note 2†)		<.001		<.001
≤30	61.8 ± 10.6		46.0 ± 16.4	
31–40	69.4 ± 9.6		53.4 ± 16.6	
41–50	68.6 ± 9.8		57.5 ± 18.9	
>50	66.4 ± 10.1		55.1 ± 18.4	
Practice				<.001
Faculty/In Practice	68.2 ± 10.0	<.001	56.6 ± 18.5	
Trainees	62.5 ± 10.6		46.9 ± 16.3	
Seen patients in last 30 days		<.001		<.001
Yes	67.6 ± 10.0		54.4 ± 18.0	
No	61.8 ± 10.9		48.6 ± 18.2	
Reside in North Carolina		.42		<.001
Yes	66.1 ± 9.6		54.6 ± 17.9	
No	65.5 ± 11.5		50.7 ± 18.4	
Use of HDS in past week	(note 3 †)	<.001		
No	63.6 ± 10.8		52.0 ± 20.2	<.001
1–3	63.7 ± 10.6		50.7 ± 19.4	
4–8	66.4 ± 10.4		52.1 ± 16.0	
≥9	70.0 ± 9.7		56.4 ± 17.3	

Note 1: Pairwise comparisons among the 5 professional groups for Knowledge: Students less than all others, P <.001. Nurses less than other professions, all P ≤ .001. Nutritionist less than Physicians, P = .02. Pairwise comparisons among the 5 professional groups for Confidence: Students less than each profession, P <.001; Nurses less than Dietitians (P =.004) and Pharmacists (P = .029); Physicians less than Dietitians, P = .03.

Note 2: Pairwise comparisons among the 4 age groups for Knowledge: ≤ 30 years old less than the other age groups, all P <.001; 31–40 and 41 – 50 greater than over 50 years old, P<0.05. Pairwise comparisons among the 4 age groups for Confidence: ≤ 30 years old less than the other age groups, all P <.001; 31–40 less than 41–50, P = .026.

Note 3: Pairwise comparisons among the 4 herb/supplement use groups for Knowledge: None and 1–3 less than 4–8 and ≥ 9, all P ≤ .003; 4–8 less than ≥ 9, P <.001. Pairwise comparisons among the 4 herb/supplement use groups for Confidence: None, 1–3, and 4–8 less than ≥9, all P ≤ .008.

*Kruskal Wallis Test for initial multiple group analysis; Mann Whitney U tests for two-group comparisons (same results obtained with T tests).

† Pairwise comparisons analyzed using Mann Whitney U tests

Overall, the mean score on the confidence scale was 52.5 (possible range of 19–95; Table 2). Confidence scores were significantly higher for pharmacists and dietitians than other professional groups; higher for those in practice than among trainees; higher for those who had seen patients in the past 30 days than those who had not; and higher for those who took nine or more HDS than those who took fewer.

All 11 items on the communication practices scale were answered by 852/853 participants who had seen a patient in the 30 days prior to enrollment. The mean score on this scale was 2.2, and scores were not normally distributed (possible range 0–10, with a peak at scores less than 3; Table 3). Communication practices scores were particularly low among students; scores were highest for those who used 9 or more HDS.

**Table 3:** Communications practicesscale scores by demographic and practice characteristics among 852 subjects who had seen patients in the past 30 days.

**Characteristic**	**N**	**Communications Practices Scale Score(Scale range 0.0 – 10.0)**	P value*
Overall mean	852	2.20 ± 1.92	
Profession (note 1†)			<.001
Physician/PA	335	2.53 ± 1.98	
Nurse	221	2.10 ± 1.93	
Nutritionist	117	2.45 ± 2.04	
Pharmacist	39	1.91 ± 1.97	
Student	140	1.45 ± 1.38	
Gender			.73
Males	224	2.12 ± 1.83	
Females	628	2.23 ± 1.96	
Age (years) (note 2†)			<.001
≤30	183	1.55 ± 1.36	
31–40	162	2.37 ± 1.92	
41–50	270	2.53 ± 2.10	
>50	237	2.22 ± 1.98	
Practice			<.001
Faculty/In Practice	600	2.41 ± 2.04	
Trainees	252	1.70 ± 1.50	
Reside in North Carolina			.001
Yes	441	1.97 ± 1.78	
No	411	2.45 ± 2.04	
Use ANY supplements (note 3 †)			<.001
No	131	1.56 ± 1.40	
1–3	276	1.83 ± 1.67	
4–8	265	2.26 ± 1.86	
≥9	180	3.16 ± 2.31	
Enrollment Period			.002
Fall	247	1.88 ± 1.72	
Spring	605	2.33 ± 1.99	

Note 1: Pairwise comparisons among the 5 professional groups: Students less than Dietitians Physicians, and Nurses, all P ≤ .006; Nurses less than Physicians P =.004; and Pharmacists less than Physicians, P = .02.

Note 2: Pairwise comparisons among the 4 age groups: ≤ 30 years old less than the other age groups, all P ≤ .003.

Note 3: Pairwise comparisons among the 4 herb/supplement use groups: None and 1–3 less than 4–8 and ≥ 9, all P ≤ .005; 4–8 less than ≥ 9, P <.001.

*Kruskal Wallis Test for initial multiple group analysis; Mann Whitney U tests for two-group comparisons.

† Pairwise comparisons analyzed using Mann Whitney U tests

### Multivariate regression analysis of predictors for the three outcomes scores (Table 4)

**Table 4:** Multivariate regression analysis of factors associated with Knowledge Test %Correct and Confidence Scale Score, and logistic regression analysis or factors associated with the Communications Practices Scale Score.

	**Variables in the Final Model for Knowledge Test (%Correct) N = 1268(Score range 0–100)**		**Variables in the Final Model for Confidence ScaleN = 1201(Scale Range 19–95)**		Variables in the Final Model for Communications Practices ScaleN = 852 *(Scale Range 0–10)	
	**Coefficient(95% CI)**	**P value**	**Coefficient(95% CI)**	**P value**	**Odds Ratio(95% CI)**	**P value**

Constant	58.4 (56.6, 60.2)	<.001	60.0 (57.8, 62.2)	<.001	Type of Health Professional‡	.013
Professional Group [Students are reference group]						
Physician/PA (yes = 1, no = 0)	6.1 (4.8, 7.5)	<.001	3.6 (1.6, 5.7)	.001	2.6 (1.3, 5.1)	.007
Nurse (yes = 1, no = 0)		NS		NS		NS
Nutritionist: (yes = 1, no = 0)	3.8 (2.1, 5.5)	<.001	5.3 (2.4, 8.1)	<.001	3.2 (1.5, 4.4)	.003
Pharmacist (yes = 1, no = 0)	6.6 (4.0, 9.1)	<.001				
						
Had seen patients in past 30 days (yes = 1, no = 0)	2.8 (1.6, 3.9)	<.001			[subjects selected on this basis]	
Enrollment period (spring = 1, fall = 0)	-2.7 (-3.9, -1.4)	<.001	-18.3 (-20.4, -16.3)	<.001		
Gender (Female = 1, Male = 0)	1.5 (0.24, 2.8)	.002				
Training Status (Faculty/In practice = 1, In Training = 0)						
Number of HDS Used in a Typical Week	0.46 (0.38, 0.54)	<.001	0.43 (0.29, 0.58)	<.001	1.11 (1.08, 1.14)	<.001
Age [age ≤ 30; represented by 0 in all other categories]						.015
Age 31–40 years (yes = 1, no = 0)	3.8 (2.3, 5.3)	<.001			2.4 (1.3, 4.4)	.006
Age 41–50 years (yes = 1, no = 0)	2.4 (1.1, 3.7)	<.001	4.8 (2.6, 5.7)	<.001	1.9 (1.0, 3.4)	.048
Age >50 years (yes = 1, no = 0)			2.4 (0.15, 4.7)	.037		
						
R-square for the final model	.251		.267		.155	

* This analysis was performed for enrollees who had seen patients in the past 30 days.

† Models were determined using backward conditional multiple regression, with P≤ .05 required for variable retention.

Several of demographic and professional characteristics were assessed in regression modeling for their relative impacts on knowledge and the confidence and communications scales: 1) type of health care professional (four 0, 1 variables representing the five categories); 2) gender; 3) age (three 0, 1 variables representing four categories); 4) whether they had seen patients in the past 30 days; 5) training status; 6) number of HDS used personally during a typical week; and 7) enrollment period (Fall vs. Spring). Age was divided into four categories because its association with the outcomes was not linear with increasing age.

#### Knowledge scores

When other demographic factors were the same, pharmacists, physicians, and dietitians answered 6.6%, 6.1% and 3.8% more of the knowledge items answered correctly than the students and nurses. The middle two age groups, having seen patients in the past 30 days, and gender also remained statistically significant. The percent correct increased by 0.46 for each herb or dietary supplement used during a typical week (e.g., an enrollee using 5 supplements would, on average, answer 2.3% more of the items correctly than an enrollee using no supplements). All P values for the final variables were =.002.

#### Confidence scale scores

Dietitians scored, on average 5.3 points higher, and physicians scored 3.6 points higher, than nurses, pharmacists and students. The two oldest age groups had higher scores than the two younger age groups. The confidence score were, on average, 0.46 units higher for a difference of one in the number of HDS used between two enrollees with otherwise identical demographic characteristics. Spring enrollees scored, on average, 18.3 units lower – or almost one Likert scale level lower per item – than Fall enrollees, even after controlling for other demographic and practice characteristics.

#### Communication scale scores

Because the Communications practices scale scores were not normally distributed, the top quartile in scores (scores greater than 3.4) was compared to the lower three quartiles. Physicians and dietitians were 2.6 and 3.2 times more likely to have scores that indicate this overall level of communications practices, or higher, than students and nurses. (Table 4) The two middle age groups (31–40 and 41–50 years old) were more likely to have upper quartile scores than those ≤30 years old. For a difference of one in number of herbs and dietary supplements used in a typical week by the enrollee, the odds ratio of being in the upper versus lower three quartiles was 1.11. A difference in use of five supplements between two individuals would yield an odds ratio of 1.7.

## Discussion

This is the largest study to date to examine expertise about HDS among diverse health professionals and trainees, and to assess factors associated with greater need for education. Despite the growth in review articles, continuing education programs and research on HDS available to interested clinicians, there is still substantial room for improvement in knowledge, confidence and communication practices in all professional groups, even in a highly self-selected group with high rates of using HDS. Our expectation that practitioners would be more confident than students was confirmed for physicians and dietitians. Our expectation that higher use of HDS would be associated with higher scores was also confirmed.

The high rate of personal use of HDS in our sample (85%) exceeds the rates reported in other studies of health professionals. For example, in a sample of 533 pharmacists in Minnesota, 53% reported personal use of HDS [22]. In a survey of dietitians, 51% reportedly consumed HDS themselves [23]. The higher rates reported in our study may be because we specifically asked about numerous commonly used vitamins and minerals as well as herbs; our respondents were also enrolling in a course to learn more about HDS. Still, we were surprised by the high median number of supplements used daily (4) in the past week. Further analyses are needed to determine factors associated with higher use of HDS among health professionals and whether higher use affects the quality of information provided to patients.

After controlling for multiple factors, knowledge scores were lowest among students and nurses, those who were not actively seeing patients, those using fewer HDS themselves and those under 31 and over 50 years old. Similar factors were associated with less confidence in communicating with patients and with poorer communication skills. Because HDS is a hot topic, we had thought that students and younger professionals might have higher scores; however, in general students and younger professionals actually had lower scores than practitioners over 30 years old. Secular trends do not easily account for the differences in knowledge scores by level of training; it is possible that clinicians acquire some education about HDS as part of practicing their profession. We were surprised that nurses as a group had lower scores on knowledge, confidence and communication practices than other professional groups. This is a new finding, and requires additional research to confirm and understand. However, students in all health professions and nurses in particular represent a prime target for curricula about HDS.

Although physicians had higher knowledge, confidence and communication practices than other professions, as a group they still had substantial room for improvement on all three outcomes, particularly for communication practices. This is consistent with previous studies. In an anonymous quiz about herbal toxicities and adverse herb-drug interactions, physicians had an average score only slightly better than that predicted by chance; better scores were not associated with age or the amount of clinical experience [24]. In a survey of physician assistants (PAs), only 19% rated their knowledge as excellent or good; 79% rated their preparation in this topic as fair to poor; those who used herbal remedies were more likely to discuss them with patients than PA's who did not use them [25]. In another physician survey, although 68% reportedly documented patients' use of non-prescription medications, only 47% documented herbal and other alternative treatments or reviewing these therapies before prescribing a new therapy [26]. In a study of 200 hospitalized patients, only 21% of the HDS used by patients (as revealed by independent personal interview and inspection of home supply) was documented in the medical record [27]. Clearly, physicians may be aware that patients are using HDS, but they are still not treating discussion about HDS in the same manner as other types of medications [28].

From our data, nurses were the professional group with the greatest need for education and training about HDS. In another survey, nurses were relatively unfamiliar with the most commonly used HDS, scoring an average of only 28% correct on a knowledge survey; the topic requiring the most improvement, as in our pilot study, was in terms of side effects and HDS-medication interactions [20,29].

Because many HDS are available in pharmacies without a prescription, shoppers may easily turn to their pharmacist for information; other health care providers may view pharmacists as experts in biochemical therapies such as HDS. In one study, on average, pharmacists reported that patients ask them questions regarding HDS 7 times per 40-hour workweek; other health care practitioners ask an average of 1.3 times per week [22].

However, the knowledge and practices of pharmacists in our sample leave substantial room for improvement, similar to a 2001 study, in which only 2.1% of pharmacists reported training specifically about herbal therapy [30]. The area of knowledge about potential HDS-medication interactions is particularly concerning. For example, in a 1999 investigation, undercover shoppers from Consumer Reports went to 25 pharmacies to buy the herb ginkgo biloba while waiting for a prescription to be filled for an anticoagulant medication. Shoppers held up both products and asked to speak with pharmacists about taking the medication. None of the pharmacists spontaneously cautioned against taking both ginkgo and warfarin concurrently; when asked directly, only 5/25 (20%) warned about potential dangers with this combination [31].

Similarly, dietitians may be seen as reliable sources of information about HDS, given the association of many HDS with dietary practices. Our results confirm a 2000 survey of 162 licensed dietitians in Oregon, in which only 10% considered themselves to be knowledgeable about HDS [32]. As with our study, another survey found greater knowledge scores among dietitians who used HDS themselves than non-users [33].

This study has several strengths and limitations. The strengths are its large sample size, the sample of diverse health professionals in practice and in training, the face validity and excellent Cronbach alpha of the study instruments. The limitations of this study include that it was a highly self-selected sample surveyed during one time period. Only 25% of respondents were male, and given women's generally greater interest in complementary therapies, gender-specific self-selection issues might limit the generalizability of these findings. Data are also based on self-report rather than direct observation or medical record review.

## Conclusion

Despite these limitations, this study offers important implications for education of health professionals and future research on clinical practice related to HDS. Clearly, clinicians of all stripes could benefit from learning more about HDS. Students appear to be a particularly ripe audience. In addition to learning about the safety, effectiveness and interactions of specific supplements, professionals require additional training to communicate with patients more consistently about these products and to document patient use and potential interactions in the medical record. Given the high prevalence of using HDS among patients and clinicians, effective educational interventions that target the unique needs of diverse professionals are urgently needed.

## Competing interests

The author(s) declare that they have no competing interests.

## Authors' contributions

KK conceived of the project, designed the survey and drafted the manuscript. PG reviewed and edited survey questions and revised the manuscript. JG marketed the project, managed enrollment and revised the manuscript. CW edited survey questions, analyzed the data and revised the manuscript. All authors read and approved the final manuscript.

## Pre-publication history

The pre-publication history for this paper can be accessed here:



## Supplementary Material

Additional File 1 The additional file (Appendix) which is in MS Word format contains the items on the Confidence and Communication Practices scales. Click here for file
