# Supplementary material for: Expertise about herbs and dietary supplements among diverse health professionals
Source: BMC Complement Altern Med. 2006 Apr 28;6:15. doi: 10.1186/1472-6882-6-15 (PMC1557537; doi:10.1186/1472-6882-6-15)
Supplement: Additional File 1 — The additional file (Appendix) which is in MS Word format contains the items on the Confidence and Communication Practices scales. [file 1472-6882-6-15-S1.doc]

**APPENDIX**

**Confidence Scale**

For each item, respondents are asked to indicate how they feel about each statement on a 5-point Likert scale including Strongly Disagree, Disagree, Neutral, Agree and Strongly Agree.

1. I feel confident responding to *patients’ questions* about H/DS.
2. I feel confident *initiating discussions* with patients about H/DS.
3. I know how to ask about which *brands and doses* patients are using of H/DS.
4. I can *warn patients* about *side effects* of commonly used H/DS.
5. I can *warn* patients about *interactions* between commonly used H/DS and medications
6. I can *provide* evidence-based information about H/DS to patients.
7. I can refer patients where to find information about the *quality* of different brands of H/DS.
8. I can tell my patients about the appropriate dose and duration to use H/DS.
9. I know where to *refer* patients for more information about H/DS
10. I know where *I* can turn for reliable information about H/DS.
11. I can readily *record* information about patients’ use of H’DS in the patient record.
12. I feel confident talking with *colleagues* about H/DS.
13. I know more about H/DS than many health care providers.
14. I know where and how to *report adverse effects* related to H/DS.
15. I could give a short lecture or demonstration to my colleagues about H/DS.
16. If a reporter or magazine writer called, I could answer questions about H/DS.
17. I can write a letter to the editor or a short review article about H/DS.
18. I can teach a high school science class about H/DS.
19. I can give a lecture about H/DS for students in my profession.

**Communication Practices Scale (answered only by participants who reported having seen a patient in the 30 days prior to survey)**

For each of the following questions, respondents were asked to estimate to the nearest 10% (from 0% to 100%). Potential responses were provided in a drop-down box on the web-based data entry screen.

1. In the past 30 days, in what percentage of your clinical encounters have you *discussed with a patient or family* the use of herbs or other dietary supplements?

2. In what percentage of these encounters did YOU initiate the discussion about herbs and supplements?

3. In the past 30 days, in what percentage of your patient encounters did you ask about the *brand name or manufacturer* of the herbs and dietary supplements used by your patients?

4. In the past 30 days, in what percentage of your patient encounters did you ask about the *dose* (amount and frequency) of herbs and dietary supplements?

5. In the past 30 days, in what percentage of your patient encounters did you ask about the *side effects* of herbs and dietary supplements used by your patients?

6. In the past 30 days, in what percentage of your patient encounters did you *provide patient handouts* or refer patients/families to specific books, articles or web sites for additional information about herbs and dietary supplements?

7. In the past 30 days, in what percentage of your patient encounters did you record the patients’ use/non-use of herbs and dietary supplements in the *patient record*?

8. In the past 30 days, in what percentage of your patient encounters in what percent of patient *records* did you note an *adverse event* from an herb or supplement?

9. In the past 30 days, in what percentage of your patient encounters did you *note an interaction* between the herb or supplement and a medication?

The last two items were Yes/No responses, scored as No = 0 and Yes = 0.5

10. In the past 30 days, have you cautioned any patient about the potential hazards associated with the use of any herbal products (other than tobacco)? __ Yes (0.5) __ No (0)

11. In the past 30 days, have you *discussed with a colleague* a clinical question related to the use of herbs or dietary supplements? __ Yes (0.5) __ No (0)
